# Clarification of Sugarcane Juice Catalyzed by Magnetic Immobilized Laccase Intensified by Alternating Magnetic Field

**DOI:** 10.3390/foods14030444

**Published:** 2025-01-29

**Authors:** Feng Wang, Mingtong Wang, Miaomiao Wang, Ling Xu, Jingya Qian, Guoqiang Guan, Baoguo Xu

**Affiliations:** School of Food and Biological Engineering, Jiangsu University, Zhenjiang 212013, China; 2222418070@stmail.ujs.edu.cn (M.W.); 2221918079@stmail.ujs.edu.cn (M.W.); lxu@ujs.edu.cn (L.X.); qianjingya@ujs.edu.cn (J.Q.); ggqyxq@ujs.edu.cn (G.G.)

**Keywords:** magnetic immobilized laccase, alternating magnetic field, sugarcane juice, clarification

## Abstract

In this study, Cu^2+^-chelated magnetic silicon dioxide nanoparticles were synthesized as carriers for laccase immobilization. The prepared magnetic immobilized laccase was applied in the clarification of sugarcane juice. The optimal conditions for the clarification of sugarcane juice with magnetic immobilized laccase in a shake flask were determined to be as follows: a temperature of 35 °C, pH of 5.5, rotation speed of 150 r/min, and immobilized laccase dosage of 1.0 mg/mL. The sucrose in the sugarcane juice inhibited both free and immobilized laccase. The inhibitory effect was characterized as mixed inhibition, wherein competitive inhibition played a dominant role. An alternating magnetic field was introduced into the catalysis process using magnetic immobilized laccase, and the catechin degradation rate was improved to 77.2% under a magnetic field intensity of 80 Gs and magnetic field frequency of 400 Hz. Under the optimal alternating magnetic field conditions, the treatment time of sugarcane juice was reduced to 20 min when catalyzed by the magnetic immobilized laccase, wherein a decolorization rate of 54.4%, reduction in turbidity of 89.7%, and total phenol degradation rate of 43.4% were achieved. Compared with the shaking condition, the assistance of alternating magnetic fields can shorten the clarifying time, increase the clarifying effect, and enhance the catalyst reusability. These results reveal useful information about the enzymatic treatment of high-sugar juice and provide a potential strategy for juice clarification with magnetic immobilized enzymes.

## 1. Introduction

Fruit juice is a kind of juice product obtained from fruits as raw materials by means of physical methods such as pressing, centrifugation, and extraction, which usually retain nutrients such as vitamins, minerals, and dietary fiber in the fruits [1,2,3]. The production process of fruit juice mainly includes raw material selection and pretreatment, crushing and pressing, filtration and clarification, blending, homogenization and degassing, sterilization and cooling, filling, and packaging steps. Juice clarification is achieved through the addition of clarifiers to fruit and vegetable juice, promoting physical–chemical reactions. These reactions cause turbid substances in the fruit and vegetable juice or certain dissolved components in the juice to precipitate. Sugarcane is rich in sugar, water, and various vitamins, fats, proteins, organic acids, calcium, iron, and other substances that are beneficial to human metabolism. Sugarcane juice, on the other hand, is the sweet juice obtained by pressing the sugarcane stems, which has the effects of clearing heat and removing toxins, quenching thirst, and supplementing nutrients. Sugarcane juice contains different phenolic compounds, including anthocyanins, catechins, gallic acid, caffeic acid, ferulic acid, and chlorogenic acid. During sugarcane juice storage, these phenolic compounds are easily oxidized by air and oxidases, leading to enzymatic browning and the formation of quinones. This results in turbidity in the sugarcane juice and affects its quality, thus worsening the consumer’s eating experience [4,5,6,7]. In addition, sugarcane juice may contain plant fibers, soil, microorganisms, and other solid impurities during the pressing process. Clarification helps to remove these insoluble and soluble impurities, improving the purity of the sugarcane juice and resulting in a smoother taste. Therefore, timely clarification of sugarcane juice is necessary.

At present, the methods of fruit juice clarification mainly include physical, chemical, and bio-enzymatic methods, among which the bio-enzymatic method has the advantages of low cost, high efficiency, and mild reaction conditions compared with the physical and chemical methods and thus has broad application prospects. The traditional methods of sugarcane juice clarification mainly include the carbonic acid method, sulfur fumigation and neutralization, etc. [8]. In recent years, scholars worldwide have also innovatively adopted a variety of new methods to solve the problem of turbidity in sugarcane juice, mainly membrane filtration technology, as well as ozone oxidation, lime, flocculation, and electro-flocculation methods. Vu et al. [9] used ultrafiltration membrane technology to reduce the color value of sugarcane juice for clarification purposes; their results indicated that a 20 nm ultrafiltration membrane removed the high-molecular-weight impurities and fine particles from the sugarcane juice, resulting in a significant reduction in the turbidity of the juice to 98.6% and achieving 50% decolorization. Ogando et al. [10] used electro-flocculation to reduce the turbidity, color, and total phenolic compounds of sugarcane juice; the color of the sugarcane juice was reduced by 73.1% after 40 min of reaction, the turbidity was reduced by 99.7%, and the total phenolic compounds were reduced by 35.2% after 1 h.

With the development of bio-enzymatic methods, more and more bio-enzymes are applied in the research of juice clarification. At present, the enzymes used for juice clarification mainly include laccase, pectinase, cellulase xylanase amylase, etc. Among them, laccase is one of the most effective enzymes used for juice clarification, and it is also environmentally friendly. In recent years, researchers have been actively applying laccase in the research of juice clarification. Immobilized laccase with transglutaminase as a cross-linking agent could provide a total phenol removal rate of 78.1% in apple juice, and the immobilized-enzyme-treated apple juice exhibited better storage stability [11]. Covalently immobilized laccase on green coconut fiber was used for clarifying apple juice, and the obtained immobilized laccase reduced the color, turbidity, and phenolic content of the juice by 61%, 29%, and 65%, respectively [12]. *Trametes versicolor* is a white-rot fungus that secretes a large amount of laccase. It is highly resistant to environmental conditions and can efficiently produce laccase under different fermentation conditions, including solid-state fermentation and liquid fermentation. In addition, *T. versicolor* can also use tea residue, agricultural residues, and other lignocellulosic wastes as substrates for laccase production, which can reduce production costs [13,14,15]. Therefore, *T. versicolor* is regarded as a good candidate for laccase production.

In this study, magnetic silica nanoparticles were used as an immobilized carrier to prepare magnetic immobilized laccase, and the optimal conditions for the clarification of sugarcane juice by this immobilized laccase were obtained. In the model system of catechins, it was judged whether sucrose had an inhibitory effect on free laccase and immobilized laccase and its specific inhibitory mechanism. An alternating magnetic field was introduced to analyze the influence of different magnetic field parameters on the catalytic properties of the enzyme and further explore whether the assistance of the alternating magnetic field could further improve the clarification effect. The inhibitory effect of sucrose in sugarcane juice on the catalytic reaction by laccase was revealed, which could be useful information for other laccase-catalyzed reaction systems. In addition, the introduction of an external alternating magnetic field accelerated the reaction catalyzed by immobilized enzymes, which exhibited good prospects in their application to enzymatic reactions in high-sugar-content systems.

## 2. Materials and Methods

### 2.1. Materials

Laccase from *Trametes versicolor* and 3-Chloropropyltrimethoxysilane (CPTS) were purchased from Sigma-Aldrich (Shanghai) Trading Co., Ltd. (Shanghai, China); sucrose, methanol (chromatographic purity), and bovine serum protein were purchased from China Pharmaceutical Group Chemical Reagent Co., Ltd. (Shanghai, China). Sugar cane was purchased from the Zhenjiang fruit wholesale market. Catechin (98%), caffeic acid, and ferulic acid were purchased from Beijing Zhongshi Qizhi Biotechnology Co., Ltd. (Beijing, China). Anthocyanin was purchased from Rometty Pharmaceuticals Durst Biology. Gallic acid, catechin, and chlorogenic acid were purchased from Beijing Solebo Technology Co., Ltd. (Beijing, China). All other materials were of analytical grade and were provided by Sinopharm Chemical Reagent Co., Ltd. (Shanghai, China). All materials were used as received without any further purification.

### 2.2. Preparation of Sugarcane Juice

Sugar cane stalks were purchased from a local market in Zhenjiang, Jiangsu Province. Sugar cane juice was collected using centrifugation after pressing it with a press machine and then centrifuged before use.

### 2.3. Preparation of Magnetic Silica Nanoparticles and Surface Modification

Fabrication and surface modification of magnetic silica nanoparticles were conducted according to our previous report [16]. Fe_3_O_4_ magnetic nanoparticles were synthesized using a chemical precipitation method. Measures of 0.99 g of FeCI_2_·4H_2_O and 2.7 g of FeCI_3_·6H_2_O were dissolved in 100 mL of ultrapure water under a N_2_ atmosphere. After heating to 80 °C, 10 mL of ammonia water was added to the solution, and the reaction lasted for 30 min under continuous agitation at 80 °C. The collected particles were washed with ultra-pure water and dispersed in ultra-pure water for use. The prepared Fe_3_O_4_ particles were coated with silicon dioxide. A mixture of 160 mL of isopropanol, 40 mL of ultra-pure water, and 5 mL of ammonia solution was mixed under intense agitation. Finally, 1 mL of tetraethylorthosilicate (TEOS) was slowly added to the dispersion under agitation, and after agitation for 12 h, the silicon dioxide was formed on the surface of Fe_3_O_4_ nanoparticles through hydrolysis and condensation of TEOS. The resulting magnetic silica nanoparticles were collected, washed with Millipore water, and dispersed in Millipore water for further use. According to the SEM results (Figure S1a), the magnetic silicon dioxide nanoparticles were spherical with an average diameter of 213 nm.

The surface of the Fe_3_O_4_-SiO_2_ nanoparticles was modified. A 2.5 mL CPTS solution was added to 100 mL of methanol aqueous solution (methanol/water = 4:1, *v*/*v*), the pH was adjusted to 4 with 0.1 mol/L hydrochloric acid, 100 mg of Fe_3_O_4_-SiO_2_ was added, and ultrasound was performed for 30 min. The recovered particles were separated with a magnet and dispersed in 100 mL of 6% iminodiacetic acid (IDA). The pH was adjusted to 9, and the reaction was stirred in a sealed container at 60 °C for 10 h. The product was repeatedly washed with ultrapure water until the pH was neutral. Magnetic particles were separated using a magnet, and then 50 mL of a 100 mg/mL CuSO_4_·5H_2_O solution was added. The reaction was carried out in a sealed container at 25 °C and 150 r/min for 1 h. The product was washed repeatedly with ultrapure water. Fe_3_O_4_-SiO_2_-CPTS-IDA-Cu^2+^ magnetically separated surface-modified magnetic particles were prepared in an aqueous solution and used as the carrier for immobilizing the laccase. An SEM image of these particles is shown in Figure S1b.

### 2.4. Laccase Immobilization

#### 2.4.1. Preparation of Immobilized Laccase

Laccase immobilization was carried out in 0.1 mol, pH 3.0 tartaric acid buffer solution. The 30 mg magnetic carrier was mixed with 15 mL of laccase solution with a protein concentration of 0.05–0.3 mg/mL in a 25 mL flask, and the laccase solution was prepared in 0.1 mol, pH 3.0 tartaric acid buffer. The resulting suspension was then incubated in a sealed container at 25 °C and 150 r/min for 2 h to achieve adsorption equilibrium. The immobilized laccase was separated by a magnet and washed with the tartaric acid buffer 2–3 times until no protein was detected in the supernatant. Finally, acetic acid–sodium acetate buffer (0.1 mol, pH 4.5) was added to prepare a magnetic immobilized laccase solution with a concentration of 1 mg/mL for later use [4,17]. SEM results of the obtained immobilized laccase are illustrated in Figure S1c. In this study, the immobilization of laccase was achieved on magnetic Cu^2+^-chelated particles by metal affinity adsorption based on non-covalent bonds. The interaction between the carrier and laccase involves establishing strong binding between the amino acid side chain group of laccase (i.e., especially imidazole groups of the histidine residues) and copper ions [17,18]. A schematic diagram of surface modification of the magnetic silica nanoparticles and laccase immobilization can be seen in Figure S2.

#### 2.4.2. Determination of Laccase Activity and Protein Content

The protein content was determined using Bradford et al.’s method [19]. The determination of laccase activity was based on the method of Wang et al. [16]. With pH 3.0 and 0.1%(*w*/*v*) catechol solution as substrate, a 100 µL laccase sample was added to a 3 mL reaction system, and the absorbance value was measured at 450 nm at 25 °C. One enzyme activity unit (U) refers to the amount of enzyme required to convert 1 µmol of substrate or form 1 µmol of product in 1 min under the above conditions. The molar extinction coefficient of pyrocatechol is 2211 L·mol^−1^ cm^−1^.

#### 2.4.3. Determination of Enzyme Activity Recovery and Immobilization Efficiency

Enzyme activity recovery and immobilization efficiency were important parameters in the process of enzyme immobilization [20]. The recovery rate of enzyme activity was calculated according to Equations (1) and (2).

(1) $$Q=\frac{V(C_{0}-C_{t})}{m}$$

Here, Q is the adsorption amount of laccase on the immobilized carrier (mg/g); C_0_ is the initial protein concentration of laccase solution (mg/mL); C_t_ is the protein concentration (mg/mL) of the supernatant after adsorption; V is the volume of the adsorption solution (mL); and m is the mass of immobilized carrier (g).

(2) $$\mathrm{AR}(\%)=\frac{A_{1}/Q}{A_{2}/C_{0}}\times 100\%$$

Here, AR is the recovery rate of laccase activity (%); A_1_ is the enzyme activity of immobilized laccase (U/g); Q is the adsorption capacity of the immobilized carrier for laccase (mg/g); A_2_ is the unit volume enzyme activity (U/mL) of free laccase solution; and C_0_ is the initial protein concentration (mg/mL) of laccase solution.

The immobilization efficiency was calculated according to Equation (3):

(3) $$\mathrm{IE}(\%)=\frac{C_{0}-C_{t}}{C_{0}}\times 100\%$$

where IE is the immobilization efficiency (%) of laccase; C_t_ is the protein concentration (mg/mL) of supernatant after adsorption; and C_0_ is the initial protein concentration (mg/mL) of laccase solution.

The relative activity was calculated in relation to the maximum activity obtained at a laccase concentration of 0.1 mg/L.

### 2.5. Effect of Reaction Conditions on Sugarcane Juice Clarification Using Immobilized Laccase

The effects of temperature, pH, rotation speed, and magnetically immobilized laccase dosage on sugarcane juice clarification catalyzed by magnetically immobilized laccase were evaluated using one-factor-at-a-time design.

In the first set of experiments, the concentration of immobilized enzyme was 0.5 mg/mL, the shaking speed was 150 r/min, the pH of sugarcane juice was 5.5, the reaction temperature was 20~40 °C, and the reaction time was 30 min. The effects of different temperatures on the clarification of sugarcane juice were investigated by measuring the turbidity, color value, and total phenol content of sugarcane juice.

In the next set of experiments, the concentration of immobilized enzyme was 0.5 mg/mL, the shaking speed was 150 r/min, the reaction temperature was 35 °C, the pH of sugarcane juice was 3.5~7.0, and the reaction time was 30 min. The effects of different pH on the clarification of sugarcane juice were investigated by measuring the turbidity, color value, and total phenol content of sugarcane juice.

In the next set of experiments, the concentration of immobilized enzyme was 0.5 mg/mL, the reaction temperature was 35 °C, the pH of sugarcane juice was 5.5, the rotation speed was 100~200 r/min, and the reaction time was 30 min. The effects of different rotation speeds on the clarification of sugarcane juice were investigated by measuring the turbidity, color value, and total phenol content of the sugarcane juice.

In the final set of experiments, the shaking speed was set to 150 r/min, the reaction temperature was 35 °C, the sugarcane juice pH was 5.5, the immobilized enzyme concentration was 0.1~1.5 mg/mL, and the reaction time was 30 min. The turbidity, color value, and total phenol content of the sugarcane juice were measured. The effects of different magnetic immobilized laccase dosages on the clarification of the sugarcane juice were investigated.

Under the optimal conditions, the changes in turbidity, color value, and total phenol content of the sugarcane juice were determined during the clarification of the sugarcane juice catalyzed by immobilized laccase, and the degradation of each monomer phenol was measured during the sugarcane juice treatment.

### 2.6. Calculation of the Initial Degradation Rate of Catechin

The catechin degradation in the initial stage (0~10 min) follows first-order chemical kinetics, as described by Equation (4):

Ln(C_t_/C_0_) = −kt (4)

where t is time and the ratio coefficient k is the initial degradation rate constant; k > 0, which does not change with the reactant content (concentration). C_t_ denotes the residual catechin concentration (mmol/L), and C_0_ denotes the initial catechin concentration (mmol/L). The amount of oxidation of catechin was calculated according to the change in absorbance and molar extinction coefficient at 390 nm. The molar extinction coefficient of catechin is 4019 L·mol^−1^ cm^−1^. In this study, catechin degradation referred to its oxidation reaction catalyzed by laccase.

### 2.7. Effect of Sugar Content on Catalytic Oxidation of Catechins by Laccase

#### 2.7.1. Catalytic Properties of Free and Immobilized Laccase

Total sugar was determined using a phenol–sulfuric acid method [21]. A series of catechin concentration reaction solutions were prepared with 0.1 mol/L pH4.0 acetic acid–sodium acetate buffer solution at different sucrose concentrations. The enzyme activity of the final laccase system was 0.01 U/mL, and the reaction was carried out at 25 °C and 150 r/min. The change in absorbance value at 390 nm within 5 min was recorded. According to the amount of catechin oxidized, the initial reaction rate was calculated, and the apparent V_max_ and apparent K_m_ of laccase at different sucrose concentrations were obtained. According to the obtained apparent V_max_ and apparent K_m_, it was fitted with the sucrose concentration. The relationship between the two and the sucrose concentration was determined, and then the Ki and Ki’ values were calculated according to the fitting equation; the Ki and Ki’ values were then compared to determine the inhibition type [22,23].

#### 2.7.2. Catechin Degradation Kinetics of Free Laccase and Immobilized Laccase

Using 0.1 mol/L pH 4.0 acetic acid–sodium acetate buffer solution as the reaction solvent system, the initial concentration of catechin was 0.8 mmol/L, and the enzyme activity of the laccase final system was 0.015 U/mL. The degradation kinetic curve of catechin under different sucrose concentrations was determined. The degradation reaction was carried out at 20 °C and 150 r/min. The changes in the catechin concentration were measured at different time points during the reaction. According to the data on the initial degradation stage and Equation (1), the initial degradation rate constant k value was obtained using linear fitting and analyzed and compared.

### 2.8. Catechin Oxidation by Immobilized Laccase Under Different Alternating Magnetic Field

According to the total sugar concentration in sugarcane juice, the sucrose concentration was set at 250 g/L; according to the optimum temperature and pH of magnetic immobilized laccase sugarcane juice treatment, the reaction temperature was set at 35 °C and the pH at 5.5. The effects of magnetic field intensity, magnetic field frequency, substrate concentration, and immobilized laccase dosage on catechin oxidation catalyzed by immobilized laccase were evaluated using one-factor-at-a-time design. The self-designed reactor for enzymatic catalysis assisted with an alternating magnetic field is shown in Figure S3.

At a sucrose concentration of 250 g/L, reaction temperature of 35 °C, pH of 5.5, magnetic field frequency of 100 Hz, and magnetic field intensity of 20–120 Gs, with immobilized laccase final system enzyme activity of 0.01 U/mL, the initial reaction rate was determined at different catechin concentrations (0.05–10 mmol/L), and the immobilized laccase apparent V_max_ and apparent K_m_ values were calculated at different magnetic field intensities. According to Equation (1), the initial degradation rate constant k values under different magnetic field strengths were calculated and analyzed. Similarly, the effect of the magnetic field frequency (75–600 Hz) on catechin oxidation was evaluated.

At a sucrose concentration of 250 g/L, reaction temperature of 35 °C, pH of 5.5, magnetic field intensity of 80 Gs, magnetic field frequency of 400 Hz, and immobilized laccase dosage of 1.0 mg/mL, the effects of the catechin concentration (0.2–3.0 mmol/L) on catechin degradation and the initial degradation rate constant were calculated and analyzed. At a sucrose concentration of 250 g/L, reaction temperature of 35 °C, pH of 5.5, magnetic field intensity of 80 Gs, magnetic field frequency of 400 Hz, and catechin concentration of 0.8 mmol/L, the effects of immobilized laccase dosage (0.2–3.0 mg/mL) on catechin degradation and the initial degradation rate constant were measured.

### 2.9. Comparison of the Catechin Degradation Rate Under Different Conditions

In order to compare the advantages of alternating magnetic fields, catechins were degraded at a sucrose concentration of 250 g/L, reaction temperature of 35 °C, immobilized laccase dosage of 1.0 mg/mL, and catechin concentration of 0.8 mmol/L; the reaction system was 50 mL. The different treatment conditions were as follows: (1) best alternating magnetic field conditions: 80 Gs and 400 Hz; (2) mechanical agitation: 150 r/min; and (3) no external magnetic field and mechanical agitation, static reaction. The catechins were sampled at 10, 30, and 60 min, and the catechin degradation rates were determined under different treatment conditions [4,24].

### 2.10. Effect of Immobilized Laccase Dosage on the Clarification of Sugarcane Juice Under an Alternating Magnetic Field

The effect of the amount of immobilized laccase (0.1–1.5 mg/mL) on the clarification of sugarcane juice was conducted at 35 °C under an alternating magnetic field of 80 Gs and 400 Hz. The color value, turbidity, and total phenol content of each sample were measured. Under the optimal conditions and under an alternating magnetic field, the changes in the color value, turbidity, and total phenol content of each sample were measured during the clarification process. The degradation of each monomer phenol before and after the treatment of sugarcane juice was determined.

To test the reusability, the sugarcane juice was clarified under the optimal conditions. After each cycle, the immobilized laccase was magnetically separated, and fresh sugarcane juice was then used to repeat the clarification operation 10 consecutive times. The residual enzyme activity was measured after each cycle, and the laccase activity after the first cycle was defined as 100%.

### 2.11. Analysis of Sugarcane Juice

The turbidity of the sample was determined by measuring the absorbance at 900 nm, and the turbidity was defined as 100 A [23]. To determine the color value [7], the Brix was measured using a refractometer, and the sugarcane juice was diluted to 1.25°Brix. The sample was filtered with a microfiltration membrane (0.45 μm), and the pH was adjusted to 7.0 with NaOH (0.05 mol/L). Then, the absorbance was measured at 420 nm with a spectrophotometer. The total phenol content was determined using the Folin–Ciocalteu method [25]. The content of different phenolic compounds in sugarcane juice was tested using the Agilent HPLC method with an AichromBcnd-AQ-C18 column (250 mm × 4.6 mm, i.d., 5 μm) and UV detector with the following conditions: flow velocity of 1.0 mL/min, injection volume of 10 μL, and mobile phase of methanol and water (7:3, *v*/*v*) [26]. The detection wavelength was 280 nm for gallic acid, catechin, and anthocyanin and 320 nm for caffeic acid, ferulic acid, and chlorogenic acid.

### 2.12. Data Analysis

All experiments were completed in triplicate. The experimental results are expressed as the mean ± standard deviation. The graphics based on the data were prepared using Origin software (OriginPro 2019b 9.6.5.169).

## 3. Results and Discussion

### 3.1. Effect of Laccase Concentration on Immobilization of Laccase on Magnetic Carrier

**Figure 1:**
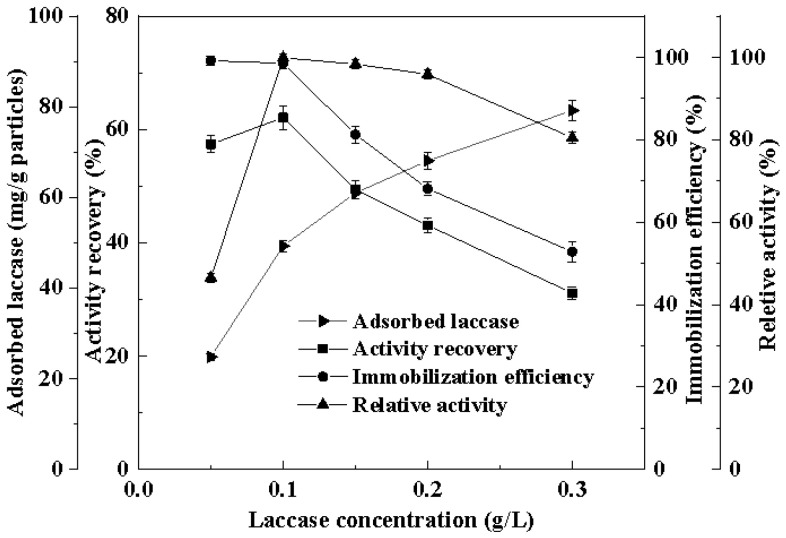
Effect of laccase concentration on laccase immobilization onto Cu^2+^-chelated magnetic silica nanoparticles.

Building on the previous research foundation regarding the effects of pH, temperature, and other factors on the immobilization of laccase on Cu^2+^-chelated silicon-based materials, the effects of different laccase concentrations on the immobilization of laccase on Cu^2+^-chelated magnetic nano-silica were investigated under the optimal immobilization conditions. The results are shown in Figure 1. When the laccase concentration was 0.1 mg/mL, the maximum activity recovery rate of 62.1% and the best immobilization efficiency of 98.6% were achieved. Under these conditions, the amount of laccase adsorbed on the Cu^2+^-chelated magnetic silica nanoparticles was 49.3 mg/g particles, showing a laccase activity of 15.3 U/g particles. The magnetic immobilized laccase obtained at the optimal laccase concentration was used for subsequent sugarcane juice clarification experiments.

### 3.2. Effect of Reaction Conditions on Sugarcane Juice Clarification with Immobilized Laccase

**Figure 2:**
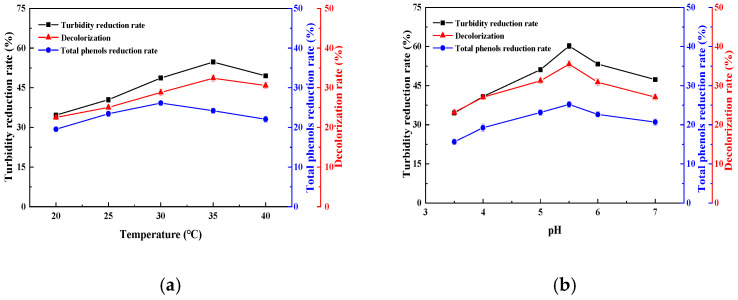

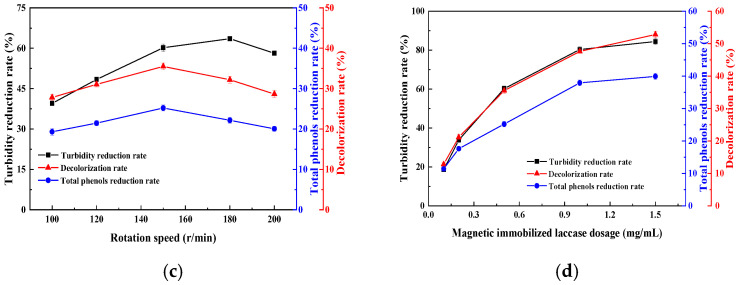
Effects of temperature (**a**), pH (**b**), rotation speed (**c**) and magnetic immobilized laccase dosage (**d**) on the clarification of sugarcane juice.

Figure 2a shows that the degradation rate of total phenols first increased and then decreased with an increase in temperature. When the temperature was 30 °C, the degradation rate of total phenols was as high as 26.2%. For the turbidity and color value, with the increase in temperature, the degradation rate also showed a trend of first increasing and then decreasing. At 35 °C, the turbidity decreased by up to 54.7% and the decolorization rate was as high as 32.4%. If the temperature is too high, the enzyme may be denatured and inactivated. If the temperature is too low, the molecular diffusion rate of the solution is slow and the catalytic efficiency is reduced. At 35 °C, the degradation rate of total phenols decreased slightly, and decreased degradation of total phenols also means that the phenols with antioxidant activity could be retained to the maximum extent while ensuring the clarification effect. Therefore, considering the degradation and clarification effect of phenols in sugarcane juice, 35 °C was selected as the best temperature for sugarcane juice clarification. Similar results were also found in the clarification of carrot–orange juice [27].

Figure 2b shows that the clarification effect of sugarcane juice was the best at pH 5.5, in which the total phenol removal rate was 25.2%, the turbidity was reduced by 60.2%, and the decolorization rate was 35.5%. This is mainly because too low or too high a pH is not suitable for the best activity of immobilized laccase, and a decrease in enzyme activity reduces the clarification effect on sugarcane juice [28,29]. When the pH is low, the amount of positive charge in the solution increases, and the insoluble suspended impurities with a negative charge in the sugarcane juice attract and polymerize each other to make the solution turbid. When the pH continues to increase near alkaline, it leads to the oxidation of fructose in the sugarcane juice and deepens the color of the solution [30]. Therefore, in this study, pH 5.5 was selected as the optimal pH for clarifying sugarcane juice; this pH is close to the actual pH value of sugarcane juice, indicating that the magnetic immobilized laccase could be directly applied to the clarification process of natural sugarcane juice.

Figure 2c shows that the clarification effect of sugarcane juice first increased and then decreased with an increase in rotational speed. When the rotation speed was 150 r/min, the total phenol degradation rate reached a maximum of 25.2%, and the color removal rate of sugarcane juice also reached a peak of 35.5%. When the rotational speed is increased, the contact collision probability between the immobilized laccase and the substrate is increased, and the catalytic efficiency of the immobilized laccase to the total phenol is increased. However, when the rotation speed is further increased, the high rotation speed causes the sugarcane juice solution to produce too high a shear force on the laccase, causing a loss of laccase activity. Thus, the total phenol degradation and decolorization rates are reduced to a certain extent. When the rotation speed was 180 r/min, the turbidity decreased most significantly to 63.6%, which was slightly higher than the 60.2% observed at 150 r/min. The main reason for this is that the fine particles in the suspension could promote the formation of clusters with an increase in rotation speed, so as to better remove them [31]. When the rotation speed continued to increase, excessive shear force was unfavorable for the stable formation of clusters and laccase activity. Therefore, in order to obtain a better clarification effect and better retain the enzyme activity of immobilized laccase, 150 r/min was selected as the optimal speed of magnetic immobilized laccase to clarify sugarcane juice in this study.

Figure 2d shows that the degradation efficiency in terms of total phenols, turbidity, and color increased with an increase in the immobilized laccase concentration. This is mainly because with an increase in the amount of magnetically immobilized laccase, the number of catalytically active sites of immobilized laccase increased and the contact probability between the substrate and laccase increased, resulting in an increase in the total phenol degradation rate and decolorization rate and a decrease in turbidity. When the dosage of magnetic immobilized laccase was 1.0 mg/mL, the total phenol degradation rate was 37.9%, the turbidity decreased by 80.3%, and the decolorization rate was 47.6%. When the dosage of magnetic immobilized laccase was 1.5 mg/mL, the total phenol degradation rate was 39.9%, the turbidity decreased by 84.4%, and the decolorization rate was 52.8%. The clarification effect increased slightly but not significantly. Guo et al. studied the effect of pectinase on juice clarification and found that the effect on juice clarification was significantly improved by increasing the amount of enzyme [32]. Therefore, considering the degradation of total phenols, clarification effect, and process economy, magnetically immobilized laccase at a concentration of 1.0 mg/mL was selected as the optimal dosage for the clarification of sugarcane juice.

Under the optimal clarification conditions, both the total phenol content and the turbidity of the sugarcane juice decreased continuously as the reaction time progressed (Figure S4). At 30 min, the turbidity of sugarcane juice decreased by 80.3%, the total phenol reduction rate was 37.9%, and the maximum color removal rate was 47.6%. The color of sugarcane juice became darker when the reaction time was further increased, which may be because, over time, catalysis results in the oxidation of small-molecular-weight polyphenols to more soluble oligomers, resulting in higher color values [4]. Therefore, the clarification time of sugarcane juice using magnetically immobilized laccase was suggested to be 30 min, wherein the best clarification effect could be achieved. The contents of anthocyanin, catechin, gallic acid, ferulic acid, and chlorogenic acid were decreased to varying degrees during the clarification process, while the contents of caffeic acid remained unchanged (Figure S5). The contents of ferulic acid, gallic acid, chlorogenic acid, anthocyanin, and catechin decreased by 94.7%, 81.5%, 73.9%, 61.1%, and 31.1%, respectively.

### 3.3. Effect of Sugar Content on Catalytic Oxidation of Catechins by Free and Immobilized Laccase

#### 3.3.1. Catalytic Properties of Free and Immobilized Laccase at Different Sucrose Concentrations

Sucrose is the main component in sugarcane juice. It is necessary to investigate the possible mechanism for its influence on laccase catalysis. Figure S6 shows that in the system without sucrose, the maximum reaction rate V_max_ of free laccase-catalyzed catechin was 1.73 μmol/L/min, and the Michaelis constant K_m_ was 0.0561 mmol/L. The maximum reaction rate V_max_ of catechin catalyzed by immobilized laccase was 4.81 μmol/L/min, which was higher than the 1.73 μmol/L/min of free laccase, but the Michaelis constant K_m_ of immobilized laccase was 0.0731 mmol/L, while the Michaelis constant K_m_ of free laccase was 0.0561 mmol/L. The increase in the V_max_ value of immobilized laccase may be due to a decrease in the toxicity of the product to the immobilized laccase, which increases the catalytic rate of immobilized laccase to catalyze catechin [33]. The increase in the K_m_ value indicates that when the immobilized laccase reaches the maximum reaction rate, the required substrate concentration increases and its affinity to the substrate is lower than that of the free enzyme. Immobilization may lead to changes in the spatial conformation of the laccase, thus decreasing the affinity to the substrate [34].

The parameters of free and immobilized lacquer enzyme kinetics at different sucrose concentrations were obtained from the fitting results of the two-reciprocal model of the Michaelis equation [35,36]. Tables S1 and S2 show that the apparent V_max_ of free and immobilized laccase decreased sequentially and the apparent K_m_ increased sequentially with increasing sucrose concentration, with the Michaelis–Menten kinetics regression curve at each sucrose concentration intersecting the second quadrant. Therefore, the inhibition of sucrose on the two laccases was characterized as mixed inhibition. The kinetic equations of mixed-type inhibition are as follows:

(5) $$V=\frac{V_{\max}\times C_{s}}{\alpha K_{m}+\alpha^{,}C_{s}}$$

derived from

(6) $$\frac{1}{V}=\frac{\alpha K_{m}}{V_{max}}\times \frac{1}{C_{s}}+\frac{1}{\frac{V_{max}}{\alpha^{\prime }}}$$

α = 1+ C_i_/K_i_′, α′ = 1 + C_i_/K_i_

αK_m_: the apparent maximum reaction rate, denoted as (V_max_)′;

$\frac{V_{max}}{\alpha^{,}}$: apparent maximum reaction rate, denoted as (V_max_)′;

C_i_: the concentration of the inhibitor;

K_i_: competition inhibition constant;

K_i_′: noncompetitive inhibition constant;

($V_{\max}$) = $\frac{V_{\max}}{\alpha^{,}}$ = $\frac{1}{1+\frac{C_{i}}{K_{i}}}\times V_{\max}$, derived from

(7) $$\frac{1}{(V_{max}{)}^{\prime }}=\frac{1}{V_{max}\times K_{i}}\times C_{i}+\frac{1}{V_{max}}$$

(K_m_)′ = αK_m_ = (1 + C_i_/K_i_′)K_m_, derived from

(8) $$(K_{m})’=K_{m}+\frac{K_{m}}{{K_{i}}^{\prime }}\times C_{i}$$

K_i_ and K_i_′ were calculated according to Equations (7) and (8), and the sizes of K_i_ and K_i_ were compared, so as to judge which inhibition was dominant in the mixed inhibition.

**Figure 3:**
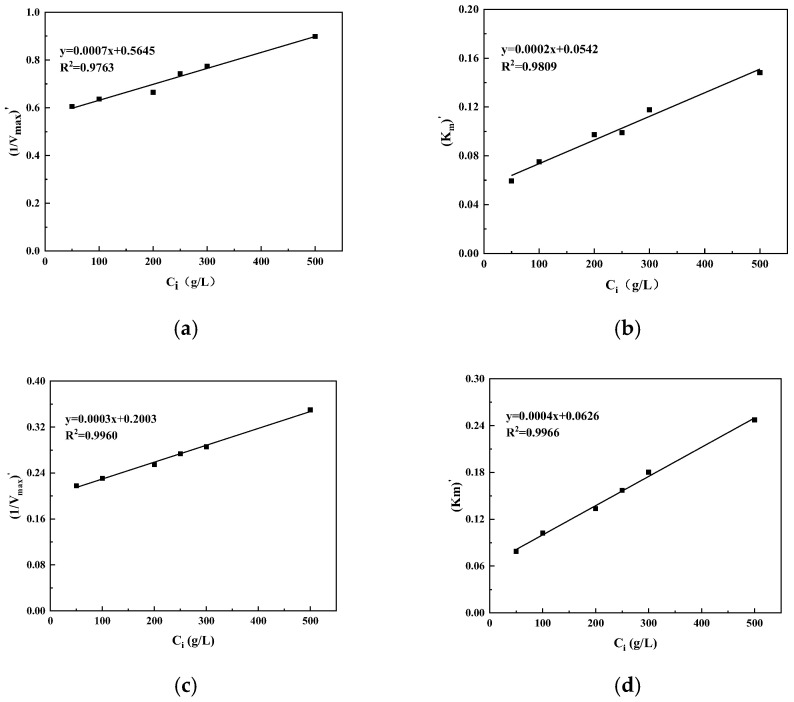
The linear relationship between sucrose concentration and apparent maximum reaction velocity (**a**,**c**) and apparent Michaelis constant (**b**,**d**) of free (**a**,**b**) and immobilized (**c**,**d**) laccase.

Figure 3 shows that the fitting regression equations between the sucrose concentration and apparent maximum reaction rate of free laccase and apparent Michaelis constant of free laccase were y = 0.0007x + 0.5645(R2 = 0.9763) and y = 0.0002x + 0.0542 (R2 = 0.9809), respectively. According to Equations (7) and (8) and the obtained fitting equation, K_i_ = 806.4 and K_i_′ = 271. Figure 3c,d show that the fitting regression equations between the sucrose concentration and apparent maximum reaction rate of immobilized laccase and apparent Michaelis constant of immobilized laccase were y = 0.0003x + 0.2003(R2 = 0.9960) and y = 0.0002x + 0.0626(R2 = 0.9966), respectively. According to Equations (7) and (8) and the obtained fitting equation, K_i_ = 667.7 and K_i_′ = 156.5. Both free laccase and immobilized laccase showed K_i_ > K_i_′, indicating that sucrose had little effect on the apparent maximum reaction rate but had a greater effect on the apparent Michaelis constant. Thus, it had a stronger effect on the binding site of laccase and substrate, mainly competitive inhibition, indicating that the inhibition mainly occurred in the binding to the substrate; that is, the inhibition mainly occurred at the catalytic site. Therefore, the inhibition effect of sucrose on these two laccases was characterized as mixed inhibition, with competitive inhibition playing a leading role.

#### 3.3.2. The Catechin Degradation Kinetics of Free Laccase and Immobilized Laccase at Different Sucrose Concentrations

**Figure 4:**
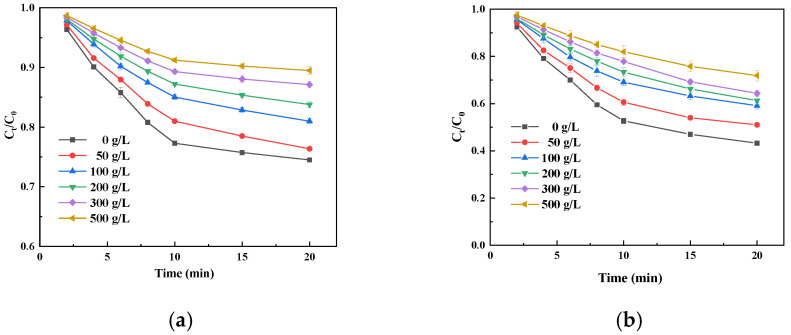

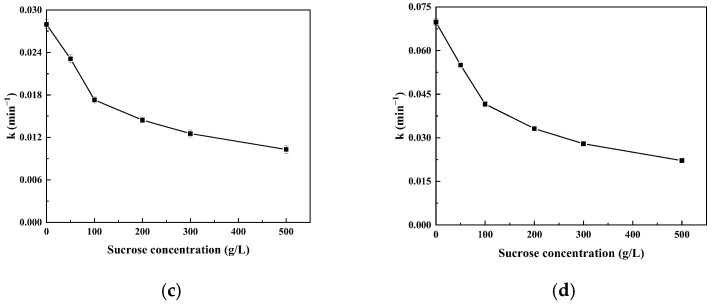
Time profile (**a**,**b**) and initial velocity constant (k; (**c**,**d**)) of catechin degradation catalyzed by free (**a**,**c**) and immobilized laccase (**b**,**d**) under different sucrose concentrations.

Figure 4 shows that with an increase in the sucrose concentration, the oxidation of catechins by free and immobilized laccase gradually decreases, which is mainly due to the inhibition of laccase activity with the increase in sucrose concentration. On the other hand, the viscosity of the solution increases with the increase in sucrose concentration, which reduces the diffusion rate of laccase and substrate, increases the mass transfer resistance, and reduces the catalytic rate of laccase [33]. Figure 4b,d indicate that the k value decreases with an increase in sucrose concentration, and the initial degradation rate of catechin by free and immobilized laccase shows a decreasing trend. These results are consistent with the changes in catechin oxidation with the sucrose concentration shown in Figure 4a,b.

### 3.4. Effect of Alternating Magnetic Field on Catechin Oxidation by Immobilized Laccase

#### 3.4.1. Effect of Magnetic Field Intensity on Catalytic Oxidation of Catechins by Immobilized Laccase

Table S3 shows that the R^2^ values of all linear regression equations are higher than 0.99, indicating that the experimental data are in good agreement with the fitting curve. When the magnetic field strength increases from 20 Gs to 80 Gs, the apparent maximum reaction rate increases and the apparent Michaelis constant decreases; more intense particle motion is achieved at higher magnetic field intensities, resulting in better mass transfer and its affinity for substrates [24,33]. However, when the magnetic field strength reaches 80 Gs, the apparent maximum reaction rate gradually decreases as the magnetic field strength continues to increase, which may be due to the further enhancement of the magnetic field strength; increased amplitudes of particle vibration (i.e., increased displacement) may exert excessive shear stress on the laccase on the magnetic carrier, leading to enzyme deactivation and a reduced catalytic effect [37]. Therefore, when the magnetic field strength is 80 Gs, the reaction rate of catechin oxidation catalyzed by magnetic immobilized laccase can be effectively increased.

**Figure 5:**
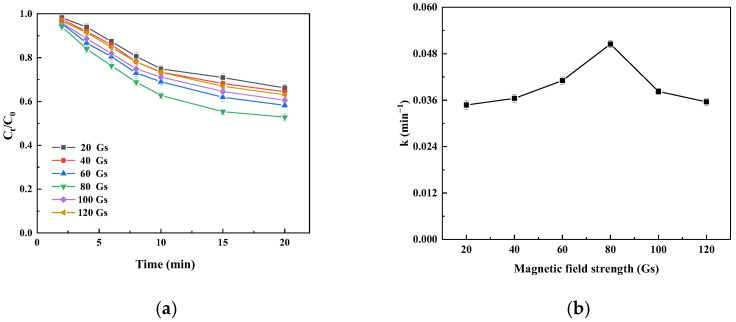
Time profile (**a**) and initial velocity constant (k; (**b**)) of catechin degradation catalyzed by immobilized laccase under different magnetic field strengths.

Figure 5a shows that the oxidation of catechins by immobilized laccase first increases and then decreases with an increase in the magnetic field intensity. This trend occurs because higher magnetic field intensity enhances the mass transfer ability and substrate affinity of immobilized laccase, which improves the catalytic effect of immobilized laccase and increases the oxidation of catechins. However, when the magnetic field strength is further increased, the vibration amplitude of immobilized laccase continues to increase, and higher magnetic field strength results in higher shear stress, which can lead to partial inactivation of immobilized laccase [37,38] and a decrease in the oxidation of catechins. Figure 5b indicates that with the increase in the magnetic field intensity, the value of K first increased and then decreased, which is consistent with the result of the change in the oxidation amount of catechin in Figure 5a.

#### 3.4.2. Effect of Magnetic Field Frequency on the Catalytic Oxidation of Catechins by Immobilized Laccase

The R^2^ values of all the linear regression equations are higher than 0.99, indicating that the experimental data are in good agreement with the fitting curve (Table S4). When the magnetic field frequency increases from 75 Hz to 400 Hz at 80 Gs, the apparent V_max_ increases and the apparent K_m_ decreases. As the magnetic field frequency increases, the alignment of magnetic nanoparticles with the magnetic field direction is prevented by the rapid change in the magnetic field direction. According to the change in the apparent Km value, the affinity of immobilized laccase to the substrate is promoted by increasing the frequency, but the effect is not as significant as that of magnetic field intensity. This is due to the excessive shear stress caused by a high frequency, leading to enzyme inactivation [37]. Therefore, an 80 Gs and 400 Hz alternating magnetic field can greatly enhance the maximum reaction rate of catechin oxidation and enzyme affinity to the substrate. The apparent V_max_ was 3.65 μmol/L/min and the apparent K_m_ was 0.1571 mmol/L in a 150 r/min shake flask (Table S1). Under the same conditions as for the other catalysts, the apparent V_max_ of immobilized laccase was 8.21 μmol/L/min and the apparent K_m_ was 0.0747 mmol/L under the condition of an alternating magnetic field of 80 Gs and 400 Hz. Therefore, the V_max_ value and its affinity to the substrate can be enhanced by an alternating magnetic field strengthening treatment, which indicates that the oscillation of magnetic particles along the magnetic field direction can enhance the mass transfer but may also weaken the inhibitive effect of macromolecular sucrose on immobilized laccase.

**Figure 6:**
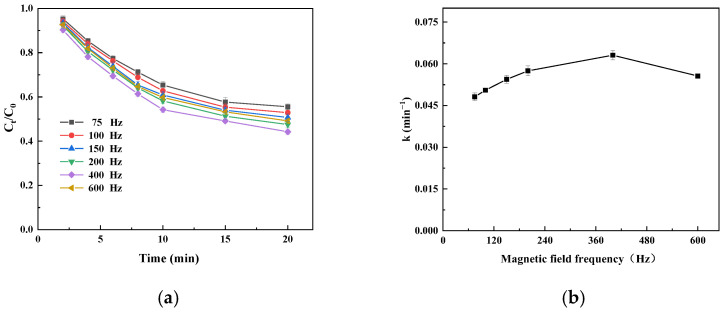
Time profile (**a**) and initial velocity constant (k; (**b**)) of catechin degradation catalyzed by immobilized laccase under different frequencies of magnetic field.

The oxidation of catechins by immobilized laccase first increased and then decreased with an increase in magnetic field frequency, reaching a maximum at 400 Hz, but when the magnetic field frequency was increased further, the oxidation of the catechins decreased; this was consistent with the variation trend in the apparent V_max_ and apparent K_m_ under different magnetic field frequencies (Figure 6a). With an increase in the magnetic field frequency, the value of k first increases and then decreases (Figure 6b). At 400 Hz, the value of k reaches its maximum; the results show that the rate of catechin degradation catalyzed by magnetic immobilized laccase could be improved by increasing the frequency of the alternating magnetic field. Under the same conditions, the degradation rate of catechin by immobilized laccase was 31.9% at 150 r/min for 20 min and 55.8% at 80 Gs and 400 Hz under an alternating magnetic field for 20 min. Therefore, the regular vibration of particles under an alternating magnetic field can bring about more effective mixing, reduce the mass transfer resistance in the reaction system, and weaken the inhibition of sucrose on immobilized laccase while reducing the toxicity of reaction products to immobilized laccase [4].

#### 3.4.3. Effect of Immobilized Laccase Dosage on Catechin Degradation Kinetics

**Figure 7:**
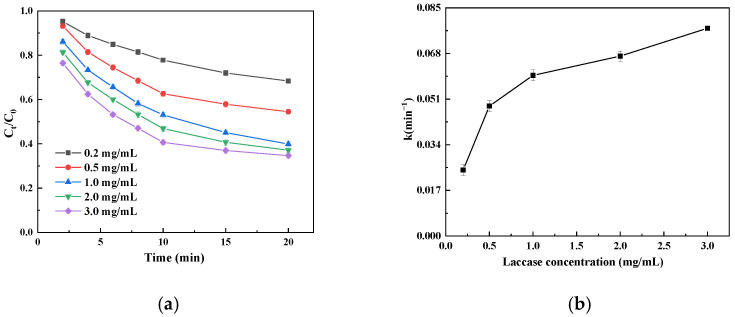
Effect of immobilized laccase dosage on catechin degradation (**a**) and the initial degradation velocity constant (k; (**b**)).

Both catechin degradation rates showed an increasing trend when the immobilized laccase amount was increased from 0.2 mg/mL to 3 mg/mL (Figure 7a); as the catechin degradation rate increased with the immobilized laccase amount, the number of catalytically active sites of catechin degradation increased. When the amount of immobilized laccase was 1 mg/mL, the catechin degradation rate was 61.1% in 20 min, while when the amount of immobilized laccase was 3 mg/mL, the catechin degradation rate was 66.2% in 20 min; this small increase in the degradation rate may be due to the decrease in the substrate concentration in the system during the middle and late stages of the reaction, and the decrease in the catalytic oxidation rate may be due to the incomplete saturation of immobilized laccase as a result of excessive dosage [38]. The k value showed an increasing trend with an increase in the amount of immobilized laccase, implying that increasing the amount of immobilized laccase is beneficial for the initial catechin degradation rate (Figure 7b). However, when the amount of immobilized laccase was increased from 0.02 mg/mL to 1.0 mg/mL, the k value changed greatly, and with a further increase in the amount of immobilized laccase, the change in the k value tended to be moderate; this trend is consistent with the results in Figure 7a. Considering the degradation rate of catechin and the economy of practical applications, the best dosage of immobilized laccase is 1 mg/mL.

#### 3.4.4. Effect of Substrate Concentration on Catechin Degradation Kinetics

**Figure 8:**
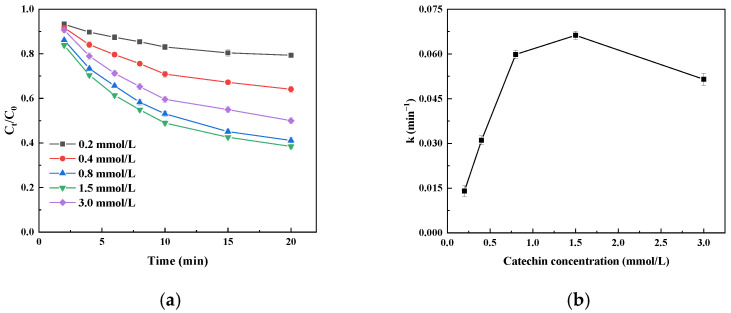
The effect of catechin concentration on its degradation (**a**) and the initial degradation velocity constant (k; (**b**)) catalyzed by immobilized laccase.

The catechin degradation rate with immobilized laccase first increased and then decreased with an increase in the catechin concentration (Figure 8a). When the catechin concentration was 1.5 mmol/L, the degradation rate was the highest; that is, C_t_/C_0_ was the lowest. This is mainly due to an increase in the initial concentration, which increases the chance of contact between the immobilized laccase and the catechin molecule. Due to the fact that the inhibition of immobilized laccase by sucrose is dominated by competitive inhibition, increasing the concentration of substrate is beneficial to releasing the inhibition of sucrose and improving the catalytic efficiency. When the catechin concentration was further increased, the catechin degradation rate decreased and C_t_/C_0_ increased, which may be because too many catechin molecules could aggregate and some molecules could not touch the active site of the immobilized laccase; more oxidation products can also produce certain toxicity against the immobilized laccase, an effect of catalytic oxidation degradation [39]. The k values tended to first increase and then decrease with increasing substrate concentration (Figure 8b), implying that the initial degradation efficiency of catechins also tends to first increase and then decrease with increasing substrate concentration; the initial degradation rate constant was maximal at a catechin concentration of 1.5 mmol/L, reaching 0.0663 min^−1^.

### 3.5. Effect of Different Mixing Conditions on Catechin Degradation Rate

**Figure 9:**
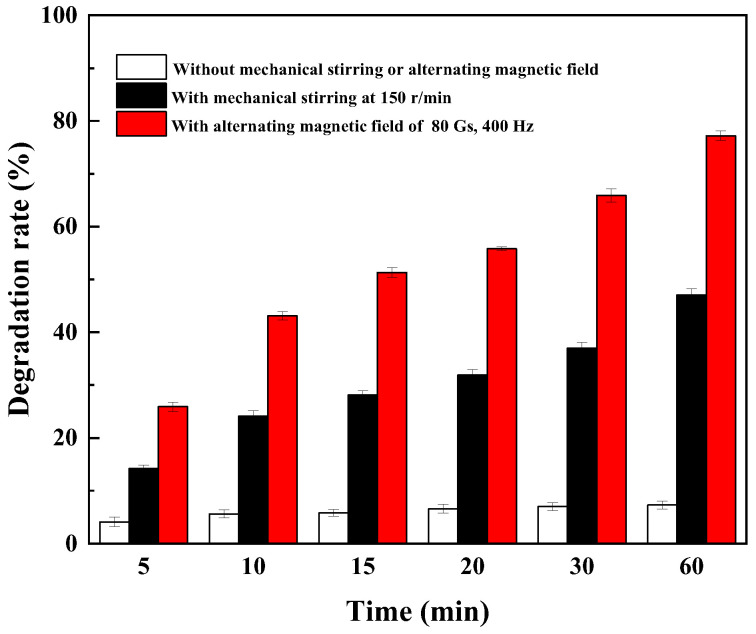
Effect of different mixed conditions on catechin degradation catalyzed by immobilized laccase.

Without mechanical agitation or an alternating magnetic field, the catechin degradation rate increased slowly and reached only 7.3% after 60 min (Figure 9). Although laccase immobilized on magnetic nano-SiO_2_ can be well dispersed in solution, its mass transfer is only accomplished by means of free diffusion, and the reaction efficiency is low; after the reaction, the degradation product forms high local concentrations around the immobilized laccase, hindering the approach to the substrate, and leads to the inhibition of enzyme activity due to product toxicity. The effect of mass transfer was improved by mechanical agitation, and the catechin degradation rate increased to 47.1% after 60 min. However, due to the high viscosity and density of the reaction system, liquid–solid two-phase synchronous motion in the reaction liquid occurs, and the mass transfer efficiency is affected. Under the condition of an alternating magnetic field, the degradation rate of catechin was the highest, reaching 77.2% in 60 min. On the one hand, the regular vibration of immobilized laccase induced by an alternating magnetic field can reduce the inhibition of sucrose and degradation products on immobilized laccase, and the apparent V_max_ increases while the K_m_ value decreases; on the other hand, the vibration of particles is equivalent to the introduction of a large number of nano–micro agitators in the system, which can effectively enhance the mass transfer in the reaction system and solve the problem of poor mass transfer in mechanical agitation and improve the reaction rate and degradation rate.

### 3.6. Effect of Immobilized Laccase Dosage on Sugarcane Juice Clarification Under an Alternating Magnetic Field

**Figure 10:**
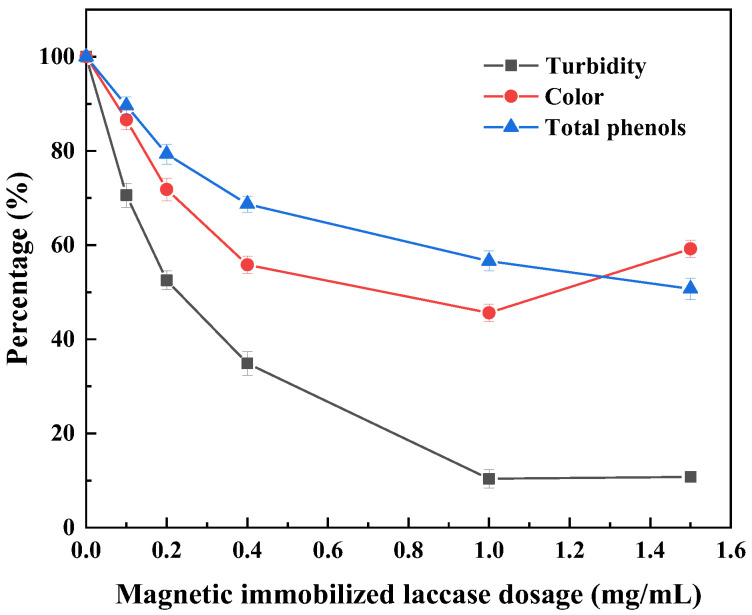
Effect of the dosage of magnetic immobilized laccase on the clarification of sugarcane juice under an alternating magnetic field.

The total phenolic compounds in sugarcane juice decrease with increasing amounts of immobilized laccase, and higher laccase amounts imply an increase in the number of catalytically active sites with an increased probability of collision with phenols. With an increase in the amount of immobilized laccase, the color value of the sugarcane juice first decreased and then increased in intensity (Figure 10). When the amount of immobilized laccase was 1 mg/mL, the color value of the sugarcane juice decreased and then increased, the decolorization rate of sugarcane juice was as high as 54.4%, and the color of the sugarcane juice increased when the amount of immobilized laccase was further increased; lower-molecular-weight phenolic compounds were further oxidized to produce more soluble oligomers, thereby increasing the color value of the sugarcane juice [4]. When the dosage of immobilized laccase was 1 mg/mL, the turbidity was the lowest, decreasing by 89.7%; the decolorization rate was 54.4%; and the total phenol degradation rate was 43.4%. Therefore, according to the clarification effect of sugarcane juice, 1 mg/mL immobilized laccase was selected as the best dosage for treating sugarcane juice.

### 3.7. Sugarcane Juice Clarification Catalyzed by Magnetic Immobilized Laccase

**Figure 11:**
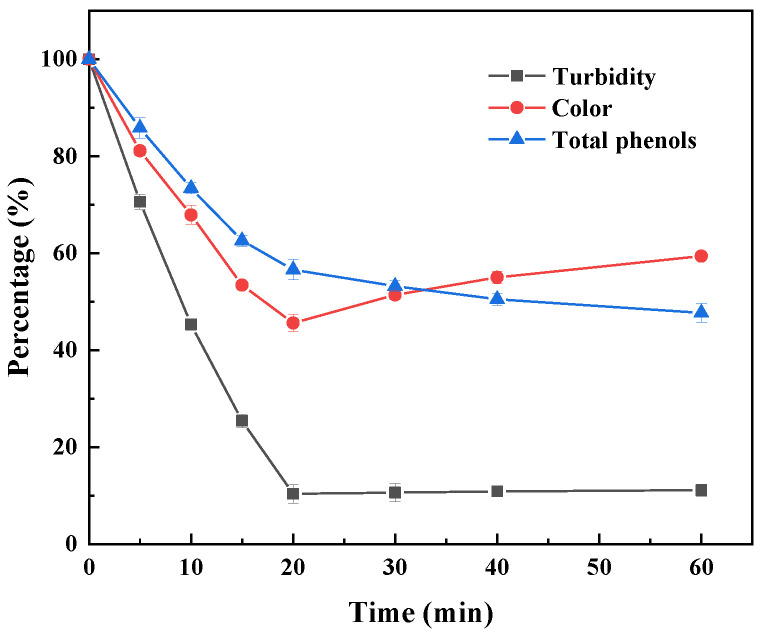
Time profile of sugarcane juice clarification catalyzed by magnetic immobilized laccase under an alternating magnetic field.

Under the condition of an alternating magnetic field, the total phenol content in the sugarcane juice decreased by 43.4% after 20 min, and the turbidity also reached the optimal treatment effect at 20 min, decreasing by 89.7% (Figure 11). With the prolongation of the treatment time, the total phenol content decreased slowly, and the turbidity did not notably decrease. The color value of the sugarcane juice reached its lowest at 20 min, decreasing by 54.4%, and a prolonged reaction time resulted in the formation of soluble oligomers, with the color value showing an upward trend. Therefore, under the optimum alternating magnetic field, the clarifying effect on the sugarcane juice was greatly improved, and the processing time was shortened from 30 min to 20 min compared to that under shaking conditions. The application of an alternating magnetic field can greatly improve the efficiency of sugarcane juice clarification, which may be due to the reduction in toxicity and space hindrance of polyphenol polymers to immobilize laccase with the help of movement under a magnetic field.

Table S5 shows the degradation rates of different phenolic compounds during clarification under an alternating magnetic field. Compared to the results under shaking conditions, the oxidation of catechin and caffeic acid slightly increased to 31.5% and 3.2%, respectively, and the oxidation of anthocyanin, gallic acid, chlorogenic acid, and ferulic acid decreased. Therefore, the application of an alternating magnetic field in sugarcane juice clarification reduced the oxidation of small-molecule monophenols, which is beneficial for maintaining the nutritional value of fruit juice [4]. However, the degradation rate of the total phenol content increased compared to that under shaking conditions, which may be due to the removal of more polyphenols, which affect the color value of sugarcane juice. Felipe et al. [10] used electro-flocculation to clarify sugarcane juice, and the content of ferulic acid was reduced by 52.1%. He et al. used the sulfite method to clarify sugarcane juice, and the contents of chlorogenic acid, gallic acid, and ferulic acid decreased by 29.3%, 38.7%, and 11.1%, respectively [40].

**Figure 12:**
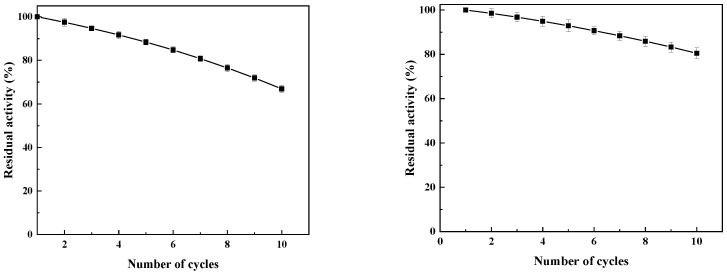
Reusability of magnetic immobilized laccase in the treatment of sugarcane juice under shaking conditions and an alternating magnetic field.

Under shaking conditions, the residual activity of the magnetic immobilized laccase was 66.9% after 10 consecutive batches of sugarcane juice clarification (Figure 12). The decrease in enzyme activity may be due to mechanical damage from prolonged processing of sugarcane juice during clarification, leading to the inactivation of immobilized laccase during recycling. In addition, the repeated use of phenolic degradation products during clarification can also inhibit enzyme activity [4]. Under an alternating magnetic field, the residual activity of the magnetic catalyst reached 80.5% of the initial enzyme activity after 10 recycling batches. Compared to the results under shaking conditions, the enzyme’s reuse performance under an alternating magnetic field was greatly improved, which may be due to the reduction in the single batch reaction time and lower inhibition of the product, resulting from the magnetic catalysts motion in the alternating magnetic field.

## 4. Conclusions

In this study, laccase was immobilized on magnetic silicon dioxide nanoparticles chelated with Cu^2+^. The enzyme activity recovery reached 62.1%, and the immobilization efficiency was 98.6%. The optimum conditions for the clarification of sugarcane juice with immobilized laccase in a shake flask were as follows: a temperature of 35 °C, pH of 5.5, rotation speed of 150 r/min, and immobilized laccase dosage of 1.0 mg/mL. Sucrose inhibited the catalytic reaction of both free and immobilized laccase in a mixed manner, where competitive inhibition was the dominant form. Under the condition of an alternating magnetic field, the best catechin degradation by immobilized laccase was achieved at 80 Gs and 400 Hz. Under the optimal conditions for the alternating magnetic field, the clarification time of the sugarcane juice was shortened to 20 min with an immobilized laccase dosage of 1 mg/mL, and the clarification indexes and catalyst reusability were greatly improved. Although magnetic silica nanoparticles have been commercialized, the surface modification of these particles is still complex. Screening simple and stable functional methods for these nanoparticles can be investigated in further studies. The alternating magnetic field showed better performance in the catalytic reaction using magnetic immobilized enzymes compared to conventional agitation. However, the cost of the apparatus may be high in large-scale applications, and an economic analysis could also be conducted in the future.

## Data Availability

The original contributions presented in this study are included in the article/Supplementary Materials. Further inquiries can be directed to the corresponding authors.

## Supplementary Materials

The following supporting information can be downloaded at: https://www.mdpi.com/article/10.3390/foods14030444/s1. Figure S1. SEM image of Fe_3_O_4_-SiO_2_ (a), Fe_3_O_4_-SiO_2_-CPTS-IDA-Cu^2+^ (b) and Fe_3_O_4_-SiO_2_-CPTS-IDA-Cu^2+^-laccase (c). Figure S2. Schematic diagram of surface modification for magnetic silica nanoparticles and its laccase immobilization. Figure S3. Diagram of self-designed reactor for enzymatic catalysis assisted by alternating magnetic field (1-water bath, 2-peristaltic pump, 3-alternating current generator, 4-coil, 5-glass reactor). Figure S4. Time profile of sugarcane juice clarification under the optimal condition (Immobilized laccase dosage 1.0 mg/mL, pH 5.5, 35 °C, 150 r/min). Figure S5. Changes in the content of phenolic compounds during the clarification of sugarcane juice catalyzed by magnetic immobilized laccase (Immobilized laccase dosage 1.0 mg/mL, pH 5.5, 35 °C, 150 r/min). Figure S6. Fitting results of Lineweaver-Burk plot of free (a) and immobilized (b) laccase under different sucrose concentration. Table S1. Catalytic kinetic parameters of free laccase at different sucrose concentrations. Table S2. Catalytic kinetic parameters of immobilized laccase at different sucrose concentrations. Table S3. Catalytic kinetic parameters of immobilized laccase at different strengths of the magnetic field. Table S4. Catalytic kinetic parameters of immobilized laccase under different frequencies of magnetic field. Table S5. The change of phenolic compounds content in sugarcane juice before and after clarification under alternating magnetic field.
