# Supplementary material for: Clarification of Sugarcane Juice Catalyzed by Magnetic Immobilized Laccase Intensified by Alternating Magnetic Field
Source: Foods. 2025 Jan 29;14(3):444. doi: 10.3390/foods14030444 (PMC11817463; doi:10.3390/foods14030444)
Supplement: Supplementary file 1 [file foods-14-00444-s001.zip › foods-3431192-supplementary.pdf]

Supplementary Materials

Figure captions

Figure S1 SEM image of Fe_3O_4-SiO_2 (a), Fe_3O_4-SiO_2-CPTS-IDA-Cu^{2+} (b) and Fe_3O_4-SiO_2-CPTS-IDA-Cu^{2+}-laccase (c).

Figure S2 Schematic diagram of surface modification for magnetic silica nanoparticles and its laccase immobilization.

Figure S3 Diagram of self-designed reactor for enzymatic catalysis assisted by alternating magnetic field (1-water bath, 2-peristaltic pump, 3-alternating current generator, 4-coil, 5-glass reactor)

Figure S4 Time profile of sugarcane juice clarification under the optimal condition (Immobilized laccase dosage 1.0 mg/mL, pH 5.5, 35°C, 150 r/min).

Figure S5 Changes in the content of phenolic compounds during the clarification of sugarcane juice catalyzed by magnetic immobilized laccase (Immobilized laccase dosage 1.0 mg/mL, pH 5.5, 35°C, 150 r/min).

Figure S6 Fitting results of Lineweaver-Burk plot of free (a) and immobilized (b) laccase under different sucrose concentration.

Wang et al., Figure S1

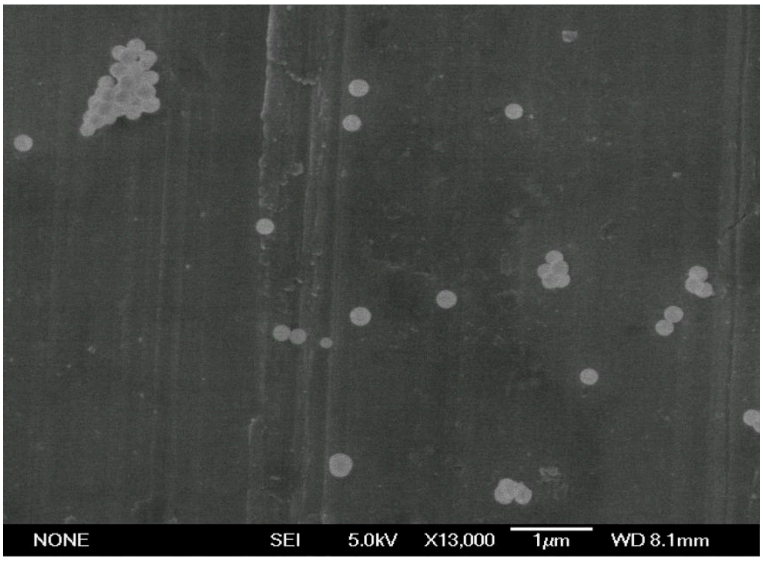

(a)

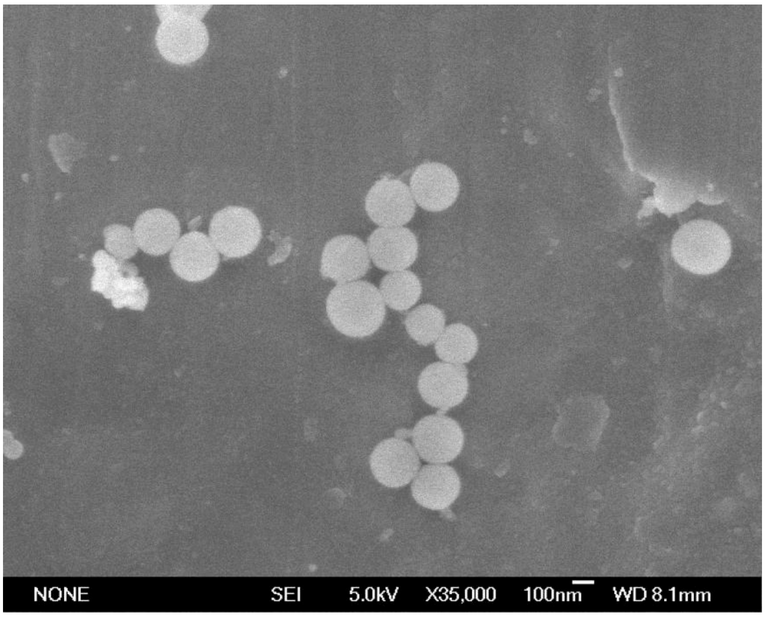

(b)

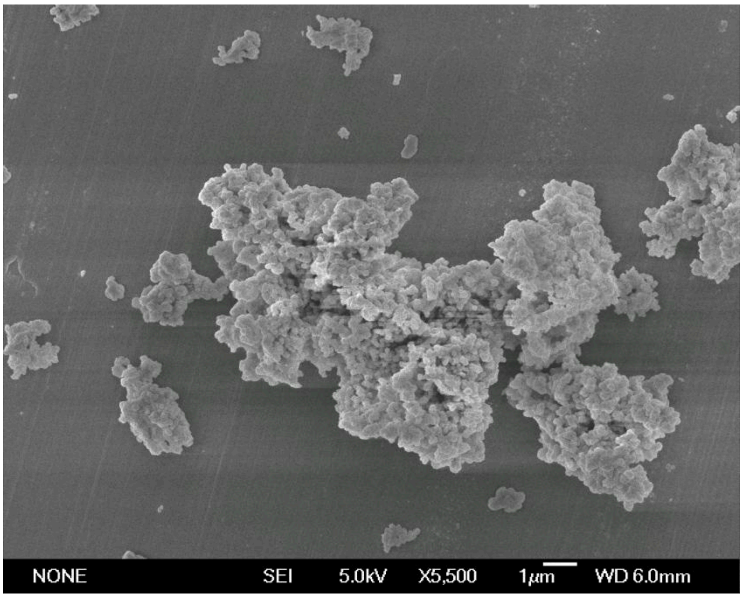

(c)

Wang et al., Figure S2

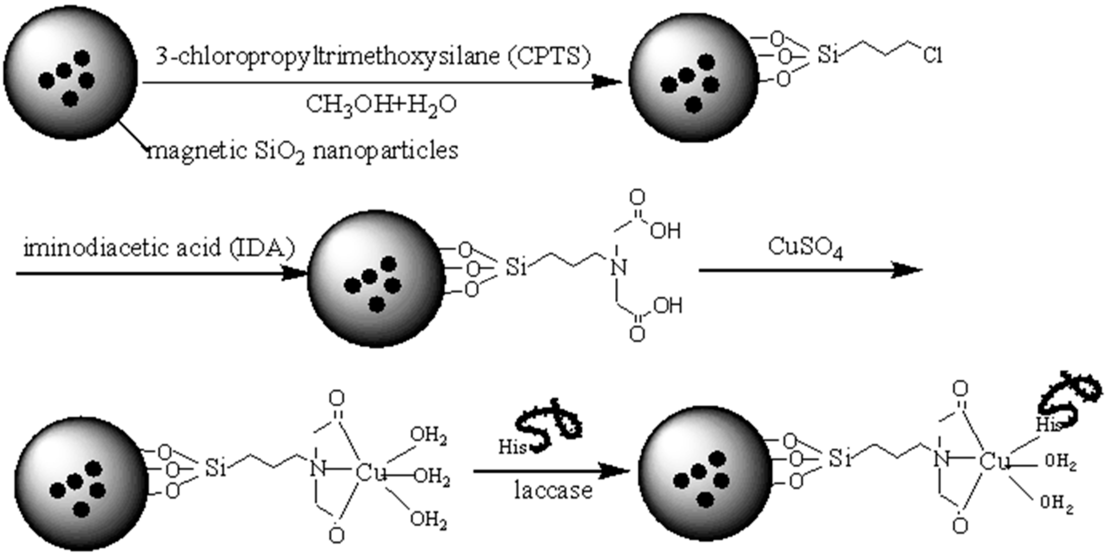

Wang et al., Figure S3

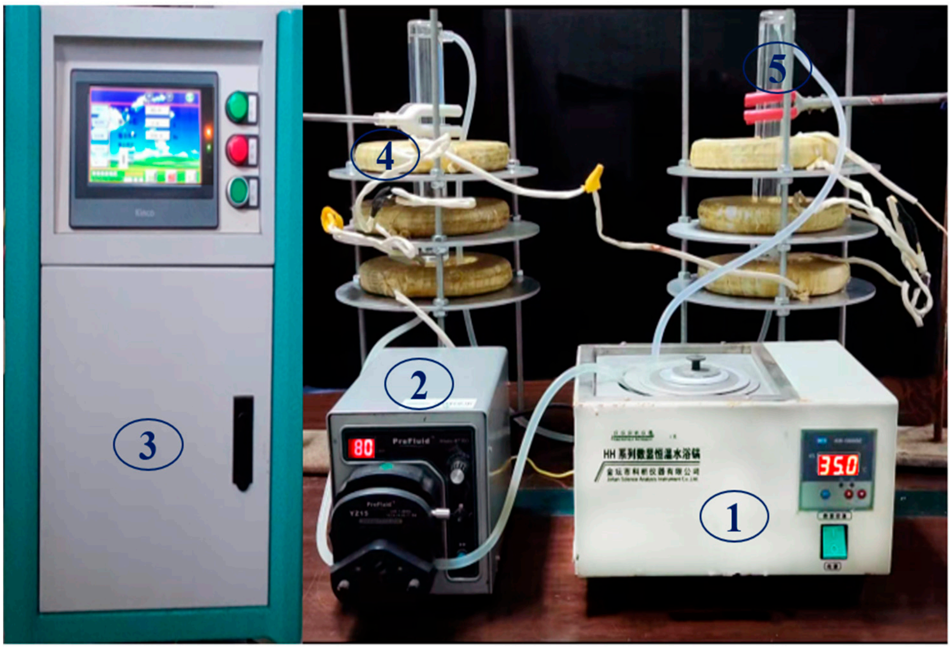

Wang et al., Figure S4

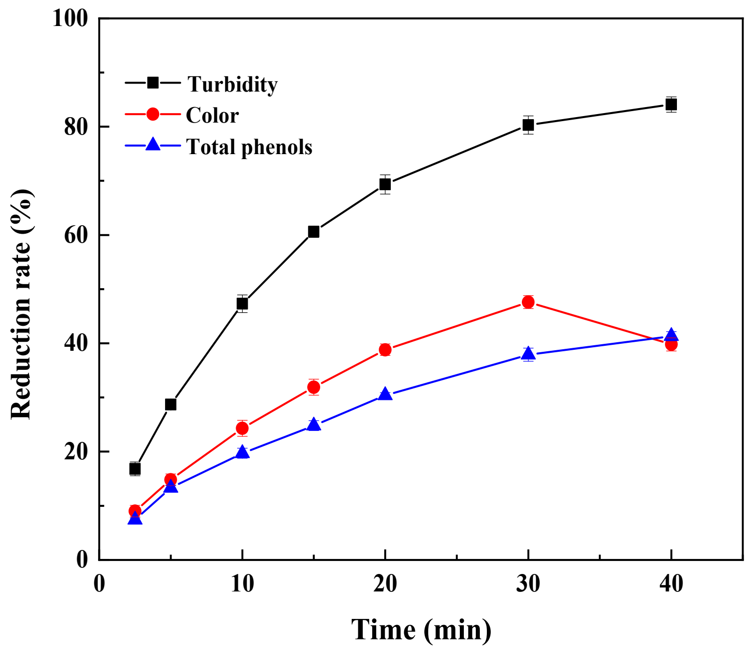

Wang et al., Figure S5

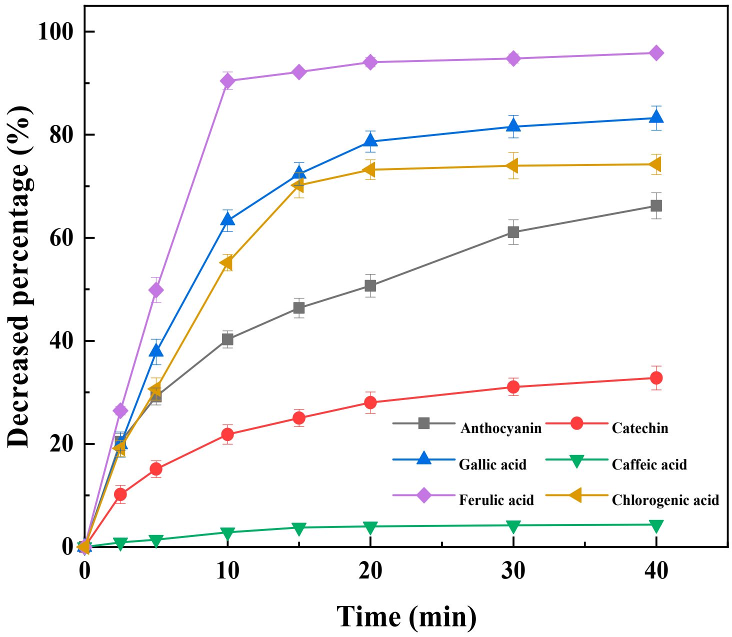

Wang et al., Figure S6

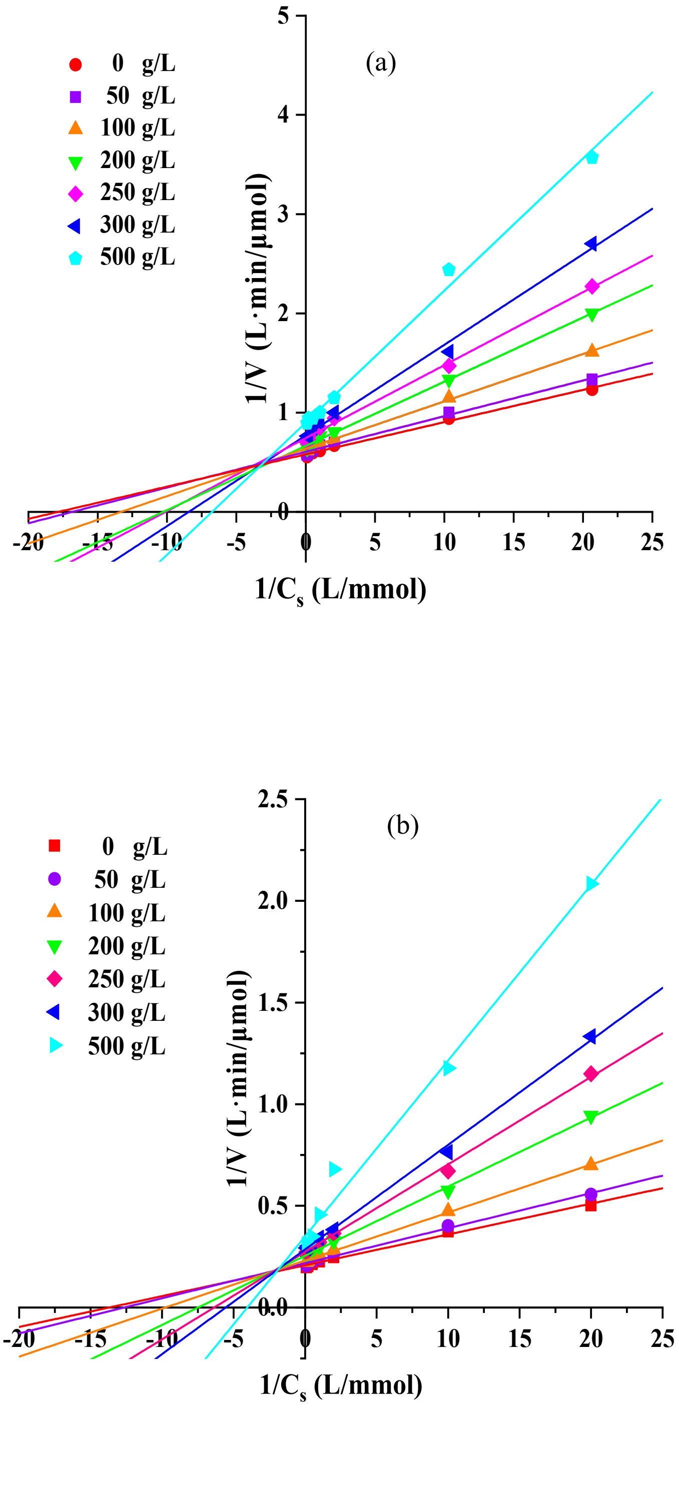

Wang et al., Table S1

Table S1 Catalytic kinetic parameters of free laccase at different sucrose concentrations.

Sucrose concentration（g/L）	1/S coefficient	Constant coefficient	Apparent V_{max}（μmol/L/min）	Apparent K_m（mmol/L）	R^2
50	0.0359±0.0012	0.6051±0.0200	1.65±0.05	0.0593±0.0022	0.9926
100	0.0478±0.0015	0.6362±0.0200	1.57±0.06	0.0751±0.0025	0.9983
200	0.0647±0.0020	0.6650±0.0227	1.50±0.04	0.0973±0.0034	0.9987
250	0.0735±0.0027	0.7430±0.0291	1.35±0.05	0.0989±0.0034	0.9958
300	0.0911±0.0031	0.7741±0.0307	1.29±0.03	0.1177±0.0042	0.9948
500	0.1331±0.0048	0.8988±0.0292	1.11±0.04	0.1481±0.0053	0.9944

Wang et al., Table S2

Table S2 Catalytic kinetic parameters of immobilized laccase at different sucrose concentrations.

Sucrose concentration（g/L）	1/S coefficient	Constant coefficient	Apparent V_{max} （μmol/L/min）	Apparent K_m （mmol/L）	R^2
50	0.0172±0.0006	0.2179±0.0072	4.59±0.16	0.0789±0.0027	0.9945
100	0.0236±0.0008	0.2309±0.0070	4.33±0.15	0.1022±0.0031	0.9987
200	0.0340±0.0013	0.2545±0.0081	3.93±0.14	0.1336±0.0049	0.9983
250	0.0430±0.0015	0.2737±0.0085	3.65±0.14	0.1571±0.0064	0.9977
300	0.0515±0.0020	0.2857±0.0094	3.50±0.13	0.1803±0.0074	0.9979
500	0.0865±0.0030	0.3498±0.0144	2.86±0.09	0.2473±0.0075	0.9870

Wang et al., Table S3

Table S3 Catalytic kinetic parameters of immobilized laccase at different strengths of the magnetic field

Magnetic field strength（Gs）	1/S coefficient	Constant coefficient	Apparent V_{max} （μmol/L/min）	Apparent K_m （mmol/L）	R^2
20	0.0387±0.0015	0.2319±0.0082	4.31±0.15	0.1669±0.0059	0.9985
40	0.0300±0.0011	0.2050±0.0077	4.88±0.17	0.1463±0.0063	0.9982
60	0.0202±0.0008	0.1997±0.0069	5.02±0.20	0.1015±0.0038	0.9965
80	0.0147±0.0005	0.1696±0.0070	5.90±0.19	0.0867±0.003	0.9989
100	0.0201±0.0007	0.2021±0.0079	4.95±0.15	0.0995±0.0031	0.9904
120	0.0239±0.0010	0.2179±0.0081	4.59±0.18	0.1097±0.0035	0.9946

Wang et al., Table S4

Table S4 Catalytic kinetic parameters of immobilized laccase under different frequencies of magnetic field.

Magnetic field frequency（Hz）	1/S coefficient	Constant coefficient	Apparent V_{max}（μmol/L/min）	Apparent K_m（mmol/L）	R^2
75	0.0164±0.0006	0.1836±0.0065	5.45±0.17	0.0893±0.0028	0.9987
100	0.0147±0.0005	0.1696±0.0061	5.90±0.20	0.0867±0.0033	0.9989
150	0.0129±0.0005	0.1517±0.0055	6.59±0.21	0.0850±0.0028	0.9991
200	0.0112±0.0004	0.1384±0.0048	7.23±0.20	0.0809±0.0034	0.9986
400	0.0091±0.0003	0.1218±0.0046	8.21±0.23	0.0747±0.0031	0.9976
600	0.0128±0.0004	0.1435±0.0052	6.97±0.19	0.0892±0.0035	0.9978

Wang et al., Table S5

Table S5 The change of phenolic compounds content in sugarcane juice before and after clarification under alternating magnetic field.

Phenolic compounds	Content before processing（mg/L）	Content after 20 min treatment（mg/L）	Degradation rate（%）
Anthocyanin	75.90±1.91	39.20±1.04	48.3±1.1
Catechin	230.08±6.01	157.60±3.62	31.5±0.7
Gallic acid	7.29±0.18	3.34±0.08	54.2±1.4
Caffeic acid	0.87±0.02	0.84±0.02	3.2±0.1
Ferulic acid	9.09±0.27	2.71±0.05	70.2±1.9
Chlorogenic acid	5.82±0.15	2.32±0.06	60.1±1.3
